# Impact of Hypertension on the Outcomes of Patients With High-Grade Gliomas: A Propensity-Matched Study From a Global Federated Health Research Network

**DOI:** 10.7759/cureus.93151

**Published:** 2025-09-24

**Authors:** Sarah Eidbo, John H Malin, Alejandro Blaubach, Alexander Pappas, Mohibur Rahman, Riley Marotta, Evan Isaacs, Akshay Ratnani, Ryan Mayo

**Affiliations:** 1 Internal Medicine, Jefferson Einstein Philadelphia Hospital, Philadelphia, USA; 2 Neurology, Jefferson Einstein Philadelphia Hospital, Philadelphia, USA; 3 Hematology and Oncology, Jefferson Einstein Philadelphia Hospital, Philadelphia, USA

**Keywords:** astrocytoma, glioblastoma multiforme, hypertension, oligodendroglioma, outcomes

## Abstract

Background: Hypertension (HTN) is known to cause endothelial dysfunction, disrupting the blood-brain barrier and cerebral blood flow. Gliomas also disrupt cerebral vasculature via tumor-associated vascular abnormalities and healthy tissue displacement. This retrospective analysis aimed to study the differences between patients diagnosed with one of the three high-grade gliomas and HTN.

Methods: We used TriNetX's US Collaborative Network to study the impact of HTN on adult patients with one of the three high-grade gliomas, namely, glioblastoma multiforme (GBM), astrocytoma, or oligodendroglioma (ODG), using ICD-10 (International Classification of Diseases) codes. We tracked these patients for five years to assess outcomes, including all-cause mortality, cerebral infarction, seizures, cerebral edema, and emergency endotracheal intubation.

Results: The GBM+HTN cohort showed significantly higher risk of all-cause mortality (HR 1.18; 95% CI 1.094-1.271; p<0.000), cerebral edema (HR 1.359; 95% CI 1.236-1.494; p<0.001), seizures (HR 1.624; 95% CI 1.459-1.808; p=0.007), and hemorrhagic stroke (HR 1.255; 95% CI 1.038-1.516; p=0.015). The ODG+HTN cohort demonstrated significantly increased risk of all-cause mortality (HR 1.398; 95% CI 1.040-1.879; p=0.001), emergency endotracheal intubation (HR 1.381; 95% CI 0.590-3.230; p=0.012), and cerebral edema (HR 0.904; 95% CI 0.680-1.203; p=0.023). There were no significant differences between the HTN and control cohorts in the astrocytoma group.

Conclusions: Patients with high-grade gliomas and HTN differ significantly. Patients with HTN appear to be at a higher risk of all-cause mortality, cerebral edema, seizures, and emergency endotracheal intubation, depending on tumor type. Further research is warranted.

## Introduction

Hypertension (HTN)

HTN affects a significant percentage of adults, with severity depending on many factors. It causes endothelial dysfunction in all areas of the body [1-3]. Within the cerebral vasculature, HTN can disrupt the blood-brain barrier, cause microvascular dysfunction, and cause neurovascular uncoupling that can disrupt cerebral blood flow [1,3,4]. Gliomas also disrupt the cerebral vasculature via tumor-associated vascular abnormalities, healthy tissue displacement, and competition for space [2]. As both HTN and gliomas appear to affect intracranial pressure (ICP), it would stand to reason that carrying both diagnoses would potentiate the effects. However, data on the topic is sparse. This retrospective analysis aimed to study the differences in outcomes between patients diagnosed with one of the three high-grade gliomas with and without HTN.

HTN and ICP

Long-standing HTN induces changes in the structure and function of the cerebral vasculature [1,3,4]. Collagen deposition and elastin fragmentation cause arterial stiffening, leading to an increased pulse pressure and vascular smooth muscle remodeling with microvascular rarefaction [1,3,5]. Perivascular space surrounding the intracerebral vasculature also enlarges, possibly affecting toxin clearance [1,3,5].

HTN may also affect cerebrovascular autoregulation, though there is limited data available in human studies [1,6,7]. Cerebrovascular autoregulation allows the body to maintain cerebral perfusion with blood pressure fluctuation [1,8]. One human study demonstrated that autoregulation may be affected in patients with untreated or malignant HTN [1,6,7]. Further studies are necessary. 

Intracerebral Tumors and ICP

The cranium is an unfortunately unique location for a tumor. Tumor sizes that would be negligible elsewhere become symptomatic and even fatal in the brain [2,9]. Tumor cells are known for their dysregulated angiogenesis from pro-angiogenic factors in the surrounding tissue that would normally be tightly regulated [2,9]. This uncontrolled angiogenesis results in immature vasculature that disrupts the blood-brain barrier, causing cerebral edema and increased ICP [2,9].

Gliomas

Most intracerebral tumors are metastases from other primary malignancies [10]. Of the primary intracerebral tumors, most are benign; only 28% of primary intracerebral tumors are malignant [10]. However, primary malignant intracerebral tumors have a poor five-year survival rate of approximately 36% [11]. Most primary malignant intracerebral tumors are one of the three types of high-grade glioma [1]. In 2021, these types were redefined by the Consortium to Inform Molecular and Practical Approaches to CNS Tumor Taxonomy-Not Official WHO (cIMPACT-NOW), which informed the World Health Organization (WHO) on modifications to glioma classification [12]. Glioma classification now incorporates molecular diagnostics among previously established clinical and histological diagnostics [12]. Three types of adult glioma are now recognized: isocitrate dehydrogenase (IDH) wild-type glioblastoma multiforme (GBM), IDH-mutant astrocytoma, and IDH-mutant and 1p/19q-codeleted oligodendroglioma (ODG) [12].

GBM

GBM is the most common primary malignant intracerebral central nervous system (CNS) tumor [11]. They are primary CNS tumors classified as WHO grade 4 histological malignancies [13]. GBMs are believed to be spontaneous in nature, but familial forms and forms associated with genetic mutations have also been described [11,14].

GBMs develop mainly within the hemispheres, brainstem, or cerebellum and only rarely metastasize [13,15-18]. They rapidly infiltrate their surroundings and become indistinguishable from healthy tissue [13,19]. GBM cells are spindle-shaped or polygonal with cell borders that are often difficult to define [13,20]. GBMs are densely over-vascularized, characteristically with necrotic foci due to insufficient blood supply from heterogeneous and inconsistent blood vessel formation [13,20,21]. These necrotic foci are often surrounded by pseudo-palisading glial cells [13,22].

The disease course is short, and resection is nearly impossible [11,19]. Chemotherapy and radiation therapy are made difficult by the blood-brain barrier and resistance of cells in hypoxic areas to radiation [13,23]. The median survival of people with tumors of this WHO grade is approximately 6-12 months [11,24].

Current mainstays of therapy include gross total resection followed by radiation and administration of alkylating agents [11,23,25]. Unfortunately, newer immune checkpoint inhibitors and molecularly targeted therapies have been proven ineffective against GBM [11,25,26]. Efforts toward developing therapies are ongoing.

Astrocytoma

Astrocytomas are another form of glioma, arising from astrocytes responsible for neuronal support, nutrient supply, repair, and immune response [27]. It is the second-most common primary intracerebral malignancy [27]. Like GBM, astrocytoma has no identified underlying cause. Its only established risk factor is previous radiation to the area. There have been associations in previous literature with conditions including Li-Fraumeni syndrome, Turcot syndrome, and neurofibromatosis type 1 [28,29].

Astrocytomas occur across all four WHO grades [29,30]. WHO grades 3 and 4 are considered high-grade and are included specifically as a category in this study [30,31].

Prognosis is poor across all WHO grades of astrocytoma [32]. For a WHO grade 4 astrocytoma, the survival time of about one year is expected [32]. Therapy focuses on quality of life and extending survival time as able [32]. Options include entire brain radiation therapy, neurosurgery, and occasionally adjuvant chemotherapy depending on the tumor grade [32].

ODG

ODGs comprise approximately 5% of primary intracranial tumors [33]. They originate from oligodendrocytes, glial cells responsible for neuronal myelination [34-36]. They are the third-most common primary intracerebral malignancy [37-39]. The majority are supratentorial, predominantly in hemispheric white matter [36]. They most often occur in the frontal lobes [35].

Although their etiology is uncertain, 70-90% have a codeletion of chromosomal arms 1p and 19q [35]. An ODG must have the 1p/19q codeletion as well as one of the two IDH mutations [37-39]. If a tumor is unable to be genetically tested but would be diagnosed as an ODG from its histology alone, it is characterized as an ODG not otherwise specified (NOS) [37-39].

Prognosis varies based on WHO grade [40]. Among high-grade ODGs, the median survival time is approximately 3.5 years [40]. Treatment options include surgery, radiation therapy, and chemotherapy, depending on the location [41]. Chemotherapy can be used alongside radiation, with ongoing trials attempting to determine the optimal regimen [42]. Monoclonal antibodies like bevacizumab have also demonstrated some benefits in anaplastic ODGs [43].

This article was previously posted to the medRxiv preprint server on April 27, 2020. 

## Materials and methods

Defining cohorts

Eligibility Criteria

This study utilized TrinetX's US Collaborative Network, a nationwide database of deidentified health data across large healthcare organizations (HCOs), to compile patients according to ICD-10 (International Classification of Diseases) codes [44]. ICD-10 codes included those related to HTN, GBM, astrocytoma, and ODG [44]. Table 1 lists the ICD-10 codes utilized to identify patients with gliomas studied.

**Table 1:** ICD-10 codes for diagnoses ICD-10 codes utilized to identify patients with the conditions studied, including HTN, GBM, astrocytoma, and ODG, are listed here. This study utilized the Kaplan-Meier analysis to calculate the log-rank test, hazard ratios, and tests for proportionality with median survival (number of days before cohort survival drops below 50%) and survival probability (percent of cohort surviving at the end of the time window). ICD: International Classification of Diseases; HTN: hypertension; GBM: glioblastoma multiforme; ODG: oligodendroglioma; NOS: not otherwise specified; IDH: isocitrate dehydrogenase

Condition	Title	ICD-10 code
HTN	Essential [primary] hypertension	ICD-10-CM I10
	Hypertensive crisis	ICD-10-CM I16
	Hypertensive urgency	ICD-10-CM I16.0
	Hypertensive emergency	ICD-10-CM I16.1
GBM	Glioblastoma, NOS	ICD-O 9440/3
	Glioblastoma IDH, wild-type [9440/3]	NAACCR 3816/5
	Glioblastoma, IDH mutant	ICD-O 9445/3
	Giant cell glioblastoma	ICD-O 9441/3
Astrocytoma	Astrocytoma, anaplastic	ICD-O 3:9401/3
	Astrocytoma, NOS	ICD-O 3:9400/3
ODG	Anaplastic oligodendroglioma IDH-mutant and 1p/19q co-deleted [9451/3]	BIOM:3816|7
	Glioma, malignant	ICD-O 3:9380/3
	Oligodendroglioma IDH-mutant and 1p/19q co-deleted [9450/3]	BIOM:3816|6
	Oligodendroglioma, anaplastic, NOS	ICD-O 3:9451/3
	Oligodendroglioma, NOS	ICD-O 3:9450/3

Cohorts Studied

For each glioma studied, two cohorts were compared: one with and without HTN as per the ICD-10 codes listed. For the GBM+HTN cohort, a query was run on 127 HCOs, with 28 providers responding and 3,992 patients matching the query criteria. For the GBM-HTN cohort, a query was run on 127 HCOs, with 29 providers responding and 5,521 patients matching the query criteria. For the astrocytoma+HTN cohort, a query was run on 142 HCOs, with 26 providers responding and 783 patients matching the query criteria. For the astrocytoma-HTN cohort, a query was run on 142 HCOs, with 32 providers responding and 3,004 patients matching the query criteria. For the ODG+HTN cohort, a query was run on 142 HCOs, with 29 providers responding and 818 patients matching the query criteria. For the ODG-HTN cohort, a query was run on 142 HCOs, with 34 providers responding and 2,628 patients matching the query criteria.

Statistical analysis

To analyze cohorts, we defined index events and time windows. The index event was the day the patient first met the selected cohort criteria. Patient outcomes, including all-cause mortality, hemorrhagic stroke, ischemic stroke, seizures, cerebral edema, intensive care unit (ICU) admission, and emergency endotracheal intubation, were followed for five years after the index event. This study utilized the Kaplan-Meier analysis to calculate the log-rank test, hazard ratios, and tests for proportionality with median survival (number of days before cohort survival drops below 50%) and survival probability (percent of cohort surviving at the end of the time window). Outcomes were defined by ICD-10 codes, including UMLS:ICD10CM:G93.6 (cerebral edema), UMLS:ICD10CM:R56 (convulsions), UMLS: ICD10CM: I63.50 (cerebral infarction), UMLS:CPT:31500 (emergency endotracheal intubation), UMLS:SNOMED:305351004 (ICU admission), and UMLS:ICD10CM:I61 (nontraumatic intracerebral brain hemorrhage) [44]. The outcome all-cause mortality was listed as a demographic. Outcomes studied are listed in Table 2.

**Table 2:** ICD-10 codes for outcomes ICD-10 codes utilized to identify patients who developed outcomes, including cerebral edema, seizures, emergency intubation, hemorrhagic or ischemic strokes, ICU admission, and mortality, are listed here. This study utilized the Kaplan-Meier analysis to calculate the log-rank test, hazard ratios, and tests for proportionality with median survival (number of days before cohort survival drops below 50%) and survival probability (percent of cohort surviving at the end of the time window). ICD: International Classification of Diseases; ICU: intensive care unit

Outcome	Title	ICD-10 code
Cerebral edema	Cerebral edema	UMLS:ICD10CM:G93.6
Seizures	Convulsions	UMLS:ICD10CM:R56
Ischemic stroke	Cerebral infarction	UMLS:ICD10CM:I63.50
Intubation	Emergency endotracheal intubation	UMLS:CPT:31500
ICU admission	Intensive care unit admission	UMLS:SNOMED:305351004
Hemorrhagic stroke	Nontraumatic intracerebral brain hemorrhage	UMLS:ICD10CM:I61
Mortality	Deceased	Deceased

This study utilized the Kaplan-Meier analysis to calculate the log-rank test, hazard ratios, and tests for proportionality with median survival (number of days before cohort survival drops below 50%) and survival probability (percent of cohort surviving at the end of the time window). Censoring was performed to account for patients who exited a cohort during the analysis time window.

Cohorts were propensity-matched according to age at index event, sex, race, ethnicity, neoplasm diagnosis, use of respiratory medications, radiology procedures, surgery, and medicine services. ICD-10 codes used for demographics include age at index event (AI), female (F), African-American (2054-5), male (M), white (2106-3), American Indian/Alaska Native (1002-5), unknown race (UNK), Native Hawaiian/Pacific Islander (2076-8), unknown ethnicity (UN), not Hispanic/Latino (2186-5), Hispanic/Latino (2135-2), other race (2131-1), and Asian (2028-9) [44]. ICD-10 codes used for diagnoses included neoplasms (C00-D49). ICD-10 codes used for procedures included radiology procedures (1010251), surgery (1003143), and medicine services/procedures (1012569) [44]. ICD-10 codes used for medications included respiratory medications (RE000) [44]. All methods were carried out in accordance with relevant guidelines and regulations. Demographics and diagnoses utilized to propensity score match cohorts are listed in Table 3. 

**Table 3:** ICD-10 codes for propensity score matching ICD-10 codes utilized for propensity score matching cohorts according to various demographics and diagnoses are listed here. This study utilized the Kaplan-Meier analysis to calculate the log-rank test, hazard ratios, and tests for proportionality with median survival (number of days before cohort survival drops below 50%) and survival probability (percent of cohort surviving at the end of the time window). ICD: International Classification of Diseases

Demographic or diagnosis	ICD-10 code
Age at index event	AI
Female	F
African-American	2054-5
Male	M
White	2106-3
American Indian/Alaska Native	1002-5
Unknown race	UNK
Native Hawaiian/Pacific Islander	2076-8
Unknown ethnicity	UNK
Not Hispanic/Latino	2186-5
Hispanic/Latino	2135-2
Other race	2131-1
Asian	2028-9
Neoplasms	C00-D49
Radiology procedures	1010251
Surgery	1003143
Medicine services/procedures	1012569
Respiratory medications	RE000

## Results

GBM

Each GBM cohort identified 2,225 patients. The average age at diagnosis for patients with HTN was 62.9±13.2 years, compared to 63.0±12.1 years for those without HTN. In the GBM+HTN cohort, 39.4% (n=877) were female, compared to the GBM-HTN cohort at 37.9% (n=843). The ethnic distribution in the HTN group was predominantly Caucasian (66.2%; n=1,473), followed by Hispanic (5.2%; n=116), Black/African American (4.7%; n=105), and Asian (1.4%; n=31). American Indian/Alaska Native and native Hawaiian/Pacific Islander each accounted for <1% of patients. In the GBM-HTN cohort, Caucasians made up 64.5% (n=1,435), followed by Hispanics (5%; n=111), Black/African Americans (4.7%; n=105), and Asians (1.2%; n=27). American Indian/Alaska Native and native Hawaiian/Pacific Islander accounted for <1%. Those of unknown race or gender were excluded from the study.

Data is represented in a format (hazard ratio, 95% CI, p-value), with a p-value equal to or less than 0.05 being considered statistically significant. The GBM+HTN cohort showed a significantly higher risk of all-cause mortality (HR 1.18; 95% CI 1.094-1.271; p<0.000), cerebral edema (HR 1.359; 95% CI 1.236-1.494; p<0.001), seizures (HR 1.624; 95% CI 1.459-1.808; p=0.007), and hemorrhagic stroke (HR 1.255; 95% CI 1.038-1.516; p=0.015). The GBM cohorts demonstrated no difference in risk of ischemic stroke (HR 2.655; 95% CI 1.678-4.199; p=0.282), emergency endotracheal intubation (HR 1.025; 95% CI 0.712-1.476; p=0.188), or ICU admission (HR 0.3; 95% CI 0.148-0.608; p=0.33). 

Astrocytoma

Each astrocytoma cohort identified 392 patients. The average age at diagnosis for patients with HTN was 50.7±15.8 years, compared to 50.1±18.0 for those without HTN. In the astrocytoma+HTN cohort, 42.9% (n=168) were female compared to 44.1% (n=173) in the astrocytoma-HTN cohort. In the astrocytoma-HTN cohort, most were Caucasian (69.6%; n=273), then Black/African American (8.9%; n=35), Hispanic (4.1%; n=16), and Asian (2.6%; n=10). American Indian/Alaska Native and native Hawaiian/Pacific Islander combined accounted for <1% of patients. In the astrocytoma+HTN cohort, patients were predominantly Caucasian (71.4%; n=280), followed by Black/African American (8.4%; n=33), Hispanic (6.1%; n=24), and Asian (2.6%; n=10). American Indian/Alaska Native and native Hawaiian/Pacific Islander accounted for <1%. Those of unknown race or gender were excluded from the study.

Data is represented in a format (hazard ratio, 95% CI, p-value), with a p-value equal to or less than 0.05 being considered statistically significant. The astrocytoma cohorts demonstrated no difference in risk of all-cause mortality (HR 1.4; 95% CI 1.104-1.775; p=0.157), ICU admission (no patients in either cohort met the outcome criteria), emergency endotracheal intubation (HR 1.703; 95% CI 0.670-4.325; p=0.422), seizures (HR 1.15; 95% CI 0.934-1.417; p=0.363), ischemic stroke (HR 1.937; 95% CI 0.863-4.347; p=0.211), hemorrhagic stroke (HR 1.47; 95% CI 0.944-2.290; p=0.087), or cerebral edema (HR 0.864; 95% CI 0.685-1.089; p=0.563). 

ODG

Each ODG cohort identified 388 patients. The average age at diagnosis for patients with HTN was 55.9±16.2 years, compared to 41.4±18.6 among those without HTN. In the ODG+HTN cohort, 41.8% (n=162) were female, compared to 47% (n=182) in the ODG-HTN cohort. In the ODG+HTN cohort, the patients were predominantly Caucasian (69.7%; n=270), followed by Black/African American (10.3%; n=40), Hispanic (4.7%; n=18), and Asian (2.3%; n=9). American Indian/Alaska Native and native Hawaiian/Pacific Islander accounted for <1%. In the ODG-HTN cohort, the patients were predominantly Caucasian (57.9%; n=225), followed by Hispanic (6%; n=23), Black/African American (5.6%; n=22), and Asian (1.8%; n=7). American Indian/Alaska Native and native Hawaiian/Pacific Islander accounted for <1%. Those of unknown race or gender were excluded from the study.

Data is represented in a format (hazard ratio, 95% CI, p-value), with a p-value equal to or less than 0.05 being considered statistically significant. The ODG+HTN cohort showed a significantly increased risk of all-cause mortality (HR 1.398; 95% CI 1.040-1.879; p=0.001), emergency endotracheal intubation (HR 1.381; 95% CI 0.590-3.230; p=0.012), and cerebral edema (HR 0.904; 95% CI 0.680-1.203; p=0.023). The ODG cohorts demonstrated no significant difference in ICU admissions (no patients in either cohort met the outcome criteria), seizures (HR 1.46; 95% CI 1.170-1.841; p=0.371), ischemic stroke (HR 3.180; 95% CI 1.165, 8.683; p=0.208), or hemorrhagic stroke (HR 1.621; 95% CI 0.966-2.720; p=0.22). 

Results are displayed in Table 4.

**Table 4:** Rates of adverse outcomes in patients with or without HTN and GBM, astrocytoma, or ODG Outcomes after propensity score matching for each comparison are displayed: GBM, astrocytoma, and ODG with and without HTN. Statistical significance is defined as a p-value equal to or less than 0.05. This study utilized the Kaplan-Meier analysis to calculate the log-rank test, hazard ratios, and tests for proportionality with median survival (number of days before cohort survival drops below 50%) and survival probability (percent of cohort surviving at the end of the time window). Data is represented in a format (hazard ratio, 95% CI, p-value). Cells with an asterisk (*) indicate a reduced risk of this outcome in the cohort without HTN. Cells with "+" indicate an increased risk of this outcome in the cohort without HTN. Cells with "-" indicate that the value could not be calculated, as no patients of either cohort met the criteria. GBM: glioblastoma multiforme; HTN: hypertension; ICU: intensive care unit; ODG: oligodendroglioma

Group 1	Group 2	Outcome	HR	95% CI	P-value
GBM without HTN	GBM with HTN	Mortality	1.18*	1.094-1.271*	0.000*
		Cerebral edema	1.359*	1.236-1.494*	0.001*
		Seizures	1.624*	1.459-1.808*	0.007*
		Ischemic stroke	2.655	1.678-4.199	0.282
		Hemorrhagic stroke	1.255*	1.038-1.516*	0.015*
		ICU admission	0.3	0.148-0.608	0.33
		Intubation	1.025	0.712-1.476	0.188
Astrocytoma without HTN	Astrocytoma with HTN	Mortality	1.4	1.104-1.775	0.157
		Cerebral edema	0.864	0.685-1.089	0.563
		Seizures	1.15	0.934-1.417	0.363
		Ischemic stroke	1.937	0.863-4.347	0.211
		Hemorrhagic stroke	1.47	0.944-2.29	0.087
		ICU admission	-	-	-
		Intubation	1.703	0.670-4.325	0.422
ODG without HTN	ODG with HTN	Mortality	1.398*	1.040-1.879*	0.001*
		Cerebral edema	0.904*	0.680-1.203*	0.023*
		Seizures	1.46	1.170-1.841	0.371
		Ischemic stroke	3.18	1.165, 8.683	0.208
		Hemorrhagic stroke	1.621	0.966-2.720	0.22
		ICU admission	-	-	-
		Intubation	1.381*	0.590-3.230*	0.012*

## Discussion

Findings

We described a nationwide retrospective analysis of patients with high-grade gliomas and HTN that demonstrated multiple significant differences compared to their counterparts without HTN. One interesting finding was a lack of significant difference between astrocytoma cohorts. Patients with astrocytoma with and without HTN had no outcome differences. Each cohort had 392 patients; while not a large number, it was larger than other glioma cohorts that did demonstrate significant differences. It is possible that the astrocytoma cohorts were too small to demonstrate significant differences, but less likely. Both astrocytoma cohorts demonstrated a 50% survival rate at the end of the window studied, which aligns with data from prior studies on prognosis [30,31]. Although it is unlikely that patients expired too quickly to produce significant results, it is still possible. The only other tumor studied with a shorter median survival time was the GBM, which still demonstrated significant differences, though with cohorts that were about five times as large. Patients with astrocytoma had low rates of emergency endotracheal intubation as well as ischemic and hemorrhagic strokes; it is possible that comorbid HTN simply did not make a difference, especially within the shorter survival time. In accordance with previous literature, seizures were common in both cohorts [30,31]. HTN could have increased seizure frequency, but due to ICD-10 coding and TriNetX database limitations, it would be impossible to tell. Cerebral edema was common in both cohorts, but the amount of edema would be difficult to compare due to similar limitations. No patients of either cohort were admitted to the ICU as per ICD-10 coding.

Both GBMs and ODGs had increased all-cause mortality when diagnosed with HTN. In recent literature, one drug used in GBM treatment, bevacizumab, appears to induce HTN [45]. However, bevacizumab-induced HTN in GBM patients has been associated with a significant survival benefit compared to normotensive GBM patients on bevacizumab [45]. These findings appear to be in conflict at first glance. Although the mechanism behind bevacizumab-induced HTN is unknown, it is theorized to be from the inhibition of the vascular endothelial growth factor (VEGF) signaling pathway, leading to increased systemic vascular resistance [45]. Patients who developed bevacizumab-induced HTN had often not previously had a HTN diagnosis, implying that the opposing outcomes may reflect different mechanisms [45,46]. This difference in outcomes may also be due to bevacizumab studies focusing on recurrent GBM management, whereas our study focused on initial GBM diagnosis [46]. Future research should aim to study bevacizumab specifically in patients with GBM and preexisting HTN.

Rates of cerebral edema were also significantly increased in patients with HTN and either GBMs or ODGs. This is likely related to microvascular proliferation rates in these tumors. As HTN increases the intravascular hydrostatic pressure, fluid is likely forced to shift into the extravascular space via the immature vasculature of each tumor. Cerebral edema is a well-known manifestation of both GBMs and ODGs; significantly higher rates with HTN are of interest [9,11,35].

Patients with GBM and HTN also had increased rates of hemorrhagic strokes and seizures. It is possible that immature tumor vasculature leads to weaker vessels that are more prone to hemorrhage when coupled with increased intravascular hydrostatic pressure from HTN [9,11,35]. Sparse information is available about any relationship between GBM and hemorrhagic stroke; only a few case reports have been published [36,37]. The association between GBMs and seizures is more well-established, with recent research postulating that neurotransmitters play a role [38,39]. The irregular tumor microenvironment is enriched with neurotransmitters that affect neuronal excitation [38,39]. Glutamate appears to have both a neuroexcitatory effect and an immunosuppressive effect that allows for tumor spread within the microenvironment [38,39]. Further studies are needed to establish any relationship.

Patients with ODG and HTN had increased rates of emergency endotracheal intubation. As ODGs are vascular tumors, it is possible that the increased emergency intubation rate is from acute tumor hemorrhage, a neurologic emergency [40]. As hypothesized previously, perhaps the increased hydrostatic pressure caused by HTN on immature microvasculature leads to more hemorrhages, thus more emergency intubations for intervention. More investigation is necessary.

Limitations

This study acknowledges several limitations. Data was collected via the TriNetX nationwide database across multiple large HCOs. TriNetX relies on ICD-10 codes. If patients were not properly coded within health records, it could lead to inappropriate inclusion or exclusion from this study. Our data is possibly affected by changes in coding or in tumor grading. The median timespan of data provided by TriNetX is 10.5 years, with some data being over 25 years old. With recent changes in tumor grading from the cIMPACT-NOW updates in 2021, this could serve as a confounder [12]. Patients could have been missed due to queries that were unable to adequately account for these changes. Results of this study should be interpreted with caution and within the appropriate context.

## Conclusions

Patients with high-grade gliomas and comorbid HTN differ significantly. Patients with HTN are at a significantly higher risk of all-cause mortality, cerebral edema, seizures, and emergency endotracheal intubation depending on the tumor type. Although patients with both GBMs and ODGs demonstrated increased rates of certain outcomes when diagnosed with HTN, this was not noted among patients with astrocytomas. Further research into these findings and into the mechanisms behind them should be explored.
